# Excessive Sedation and Catatonia-Like Presentation From Risperidone-Valproate Interaction: A Case Report

**DOI:** 10.7759/cureus.108368

**Published:** 2026-05-06

**Authors:** Da Young Lee, Teresa Canova, Kebria Kashfi, Jessica V Ojeda, Celia M Canova, Rukhsar Shaikh

**Affiliations:** 1 School of Medicine, Florida International University Herbert Wertheim College of Medicine, Miami, USA; 2 Department of Psychiatry, Southern Winds Hospital, Hialeah, USA; 3 School of Medicine, Ross University School of Medicine, Bridgetown, BRB; 4 Department of Graduate Medical Education, Jackson Memorial Hospital, Miami, USA; 5 School of Medicine, St. George's University School of Medicine, St. George's, GRD; 6 School of Medicine, American University of Antigua College of Medicine, Osbourn, ATG

**Keywords:** bipolar disorder, catatonia-like syndrome, central nervous system depression, drug-drug interaction, medication-induced immobility, olanzapine, psychiatric inpatient, risperidone, sedation, valproate

## Abstract

Sedation is a known adverse effect of risperidone, but catatonia-like immobility potentiated by valproate is rarely reported. A 23-year-old woman with bipolar disorder, admitted for acute psychosis, was treated with risperidone titrated to 3 mg twice daily alongside valproate. She developed escalating somnolence, immobility, refusal of food, and tremors. Laboratory evaluation revealed a supratherapeutic valproate level (125 µg/mL); medical workup and a lorazepam challenge were unrevealing. Discontinuation of risperidone with substitution with olanzapine resulted in rapid and complete recovery within 24 hours. This case highlights the risk of severe catatonia-like sedation from a risperidone-valproate interaction. It underscores the importance of recognizing medication-induced causes of catatonia-like presentations to enable timely intervention.

## Introduction

Risperidone, a widely prescribed second-generation antipsychotic, is a mainstay in the treatment of schizophrenia, schizoaffective disorder, and acute manic or mixed episodes of bipolar disorder due to its potent dopamine D2 receptor antagonism and well-established efficacy in controlling positive psychotic symptoms. Its adverse effect profile encompasses a range of metabolic and neuropsychiatric changes. The most frequent adverse effects of intramuscular risperidone in patients with schizophrenia (≥5%) include headache, parkinsonism, dizziness, akathisia, fatigue, constipation, dyspepsia, sedation, weight gain, extremity pain, and dry mouth [1]. In patients with bipolar disorder, the most common adverse effects include weight gain (5% in a monotherapy trial) and tremor and parkinsonism (≥10% in an adjunctive therapy trial). Although sedation is a well-recognized effect, the development of profound immobility, mutism, and refusal to eat resembling catatonia is highly unusual [2]. Such presentations can complicate the diagnostic process, particularly in inpatient psychiatric settings, where catatonia itself is not uncommon [3].

Catatonia is a complex neuropsychiatric syndrome defined in the Diagnostic and Statistical Manual of Mental Disorders, Fifth Edition, Text Revision, by abnormal behavior and motor signs, including catalepsy, purposeless motor activity, posturing, negativism, mutism, and refusal to eat. It occurs in approximately 9-17% of psychiatric inpatients. Its etiologies are diverse, ranging from primary psychiatric disorders (e.g., schizophrenia and mood disorders) to medical conditions and medication-induced states [4]. Benzodiazepines, particularly lorazepam, are considered first-line treatment for primary catatonia, whereas management of drug-induced cases focuses on identifying and discontinuing the offending agent [5].

Valproate is frequently used as a first-line mood stabilizer for bipolar disorder and is generally well tolerated at therapeutic serum concentrations. However, at supratherapeutic levels, it can cause CNS depression, with reported adverse effects including tremor, confusion, sedation, and encephalopathy [6].

This report describes a young woman who developed rapid-onset, severe immobility and refusal to eat while receiving both risperidone and supratherapeutic valproate. Her symptoms resolved almost immediately after discontinuation of risperidone and substitution with olanzapine. This case raises concern for a potential pharmacokinetic interaction between risperidone and valproate, resulting in catatonia-like features, and underscores the need for clinical vigilance when co-prescribing these agents.

## Case presentation

A 23-year-old woman with a history of bipolar disorder was admitted to the inpatient psychiatric unit for acute psychosis characterized by paranoia, auditory hallucinations, and disorganized thought processes. At baseline, she is a young adult who is intermittently noncompliant with prescribed medications; when off medication, she typically develops command auditory hallucinations but is not somnolent or withdrawn. She had several prior psychiatric admissions and had been prescribed valproate as a mood stabilizer. She had no significant medical comorbidities, no history of substance use, and no other chronic illnesses.

On admission, risperidone was initiated at 1 mg twice daily and titrated over several days to 3 mg twice daily due to persistent psychotic symptoms. She continued valproate for mood stabilization. Subsequent laboratory testing revealed a serum valproate level of 125 µg/mL (therapeutic range: 50-100 µg/mL). As the risperidone dose was escalated, the patient developed progressive somnolence, coarse tremors, groaning, and refusal to eat and, ultimately, became bedridden. She required one-to-one observation due to an inability to perform activities of daily living; nursing staff reported that she required assistance with feeding and hygiene. Vital signs remained stable. Laboratory evaluations, including complete blood count, comprehensive metabolic panel, thyroid function tests, serum ammonia level, urinalysis, electroencephalography, and head CT, were unremarkable aside from the supratherapeutic valproate level.

The sequence of clinical events, medication adjustments, and patient outcomes during hospitalization is summarized in Table 1.

**Table 1:** Chronological summary of clinical events, medication changes, and observed outcomes during the patient’s hospitalization

Day	Clinical event	Outcome
Day 0	Admission for acute psychosis	Baseline psychosis symptoms: paranoia, hallucinations
Days 1-3	Risperidone initiated at 1 mg BID, valproate continued	Initial mild improvement in psychosis; patient ambulatory
Day 4	Risperidone titrated to 3 mg BID	Increased sedation observed
Day 5	Somnolence, tremors, refusal to eat; 1:1 observation started	Catatonia-like immobility; no response to lorazepam
Day 6	Valproate level: 125 µg/mL; labs and CT unremarkable	No acute medical findings; drug interaction suspected
Day 7	Risperidone stopped; olanzapine 5 mg started	Rapid clinical improvement within 24 hours

The differential diagnosis included primary catatonia, neuroleptic malignant syndrome, medication-induced immobility, valproate toxicity, metabolic derangements, and infectious etiologies. Catatonia was initially suspected due to immobility and refusal to eat; however, there was no autonomic instability, and a lorazepam challenge produced no improvement, arguing against primary catatonia. Neuroleptic malignant syndrome was considered less likely because there was no rigidity, hyperthermia, or autonomic instability. The supratherapeutic valproate level, in conjunction with recent risperidone titration, supported a drug-induced etiology [7,8]. Despite repeated transfers for medical clearance, all evaluations ruled out acute medical causes. The lack of response to lorazepam further emphasized the need to evaluate for medication-related toxicity.

Given the lack of response to lorazepam and repeated negative medical evaluations, risperidone was discontinued. The patient was started on olanzapine 5 mg nightly, a regimen less influenced by valproate’s inhibitory effects. Within 12 hours of the medication change, she was able to get out of bed and shower independently. By 24 hours, she was ambulating on the unit, eating meals, and described herself as “much clearer.” Her tremors resolved completely, and she actively engaged in psychiatric treatment without further episodes of immobility.

## Discussion

This case illustrates the diagnostic challenge of distinguishing medication-induced catatonia-like syndromes from primary catatonia. Both risperidone and valproate can cause sedation; however, when combined, particularly in the setting of supratherapeutic valproate levels, their CNS depressant effects may be amplified. This can lead to profound immobility and refusal to eat that closely resemble catatonia. Such iatrogenic presentations are easily misattributed to an exacerbation of underlying psychiatric illness [6].

To better understand the clinical outcome in this case, it is helpful to consider existing comparative data on risperidone and olanzapine. Risperidone is metabolized primarily by CYP2D6, and its active metabolite, paliperidone, may contribute to sedative effects [9]. Valproate inhibits hepatic metabolic pathways, including glucuronidation and CYP2C9, which may increase risperidone exposure and potentiate CNS depression [6]. This interaction may have contributed to the patient’s profound sedation, tremors, and catatonia-like immobility [10-12]. In contrast, olanzapine is metabolized primarily by CYP1A2 and UGT1A4, pathways less affected by valproate [1,13].

Notably, risperidone is not generally associated with a higher risk of extrapyramidal symptoms compared to olanzapine. Rather, its interaction with valproate appears more likely to potentiate CNS adverse effects. A randomized, double-blind trial comparing risperidone and olanzapine in patients with schizophrenia or schizoaffective disorder found both medications to be efficacious and similarly well tolerated, with no significant difference in extrapyramidal symptoms. Among study completers, risperidone produced greater reductions in positive and affective symptoms, whereas olanzapine was associated with greater weight gain; importantly, olanzapine was not superior to risperidone in any domain [7,14]. These findings suggest that the catatonia-like syndrome observed in our patient reflects a specific pharmacokinetic interaction between risperidone and valproate, rather than an intrinsic susceptibility to extrapyramidal effects from risperidone. This case contributes to the limited literature on interactions between antipsychotics and mood stabilizers and highlights the importance of vigilant monitoring for unexpected adverse effects.

While sedation is among the most common adverse effects of antipsychotics, the development of catatonia-like immobility is rare. Catatonia itself occurs in approximately 5-20% of acute psychiatric inpatients [15]; however, medication-induced catatonia-like presentations remain underrecognized and likely underreported [14]. Because valproate is widely used in bipolar disorder and risperidone is a commonly prescribed first-line antipsychotic, this adverse interaction, although uncommon, may be encountered more frequently than is currently reflected in the literature.

**Figure 1:**
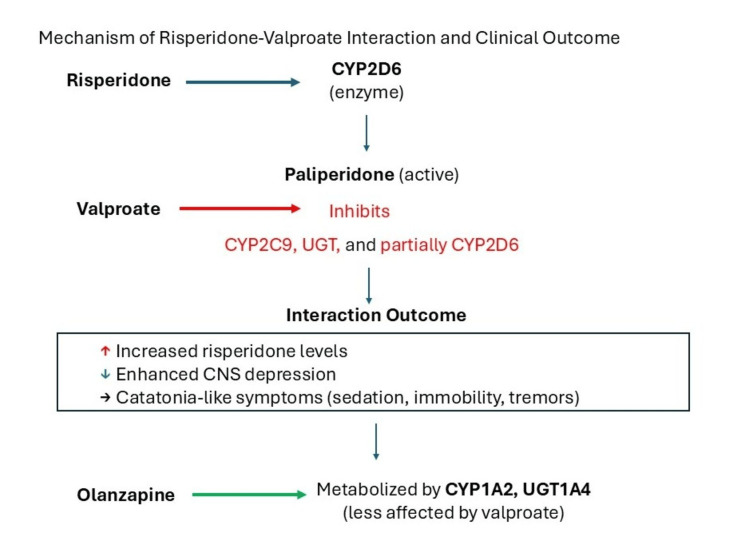
Potential pharmacokinetic interaction between risperidone and valproate highlighting the inhibitory effects on metabolism and the clinical outcome of sedation and immobility

The proposed interaction between risperidone and valproate, contributing to CNS depression and immobility, is illustrated in Figure 1.

Misdiagnosis of medication-induced immobility as primary catatonia can have serious consequences. Patients may undergo unnecessary medical transfers, repeated laboratory and imaging studies, and benzodiazepine trials without clinical benefit. Such delays can prolong hospitalization, and, in severe cases, food refusal and immobility may lead to dehydration, malnutrition, and other secondary complications.

The prognosis for drug-induced catatonia-like syndromes is generally favorable once the offending medication is identified and discontinued. In our patient, switching from risperidone to olanzapine, which is metabolized primarily by CYP1A2 and UGT1A4 and is therefore less affected by valproate, led to rapid clinical improvement. She regained the ability to shower within 12 hours of the medication change and was ambulating on the unit within 24 hours.

Important ethical considerations also arise in such cases. Patients with catatonia-like immobility are often unable to provide a reliable history or participate meaningfully in decision-making. Clinicians must balance the need for thorough medical evaluation with the obligation to avoid unnecessary procedures and transfers. Medication-induced catatonia-like presentations require a different therapeutic approach than primary catatonia, and early recognition is essential. Prompt identification can prevent unnecessary medical transfers, repeated diagnostic workups, and ineffective treatments. In this case, substituting olanzapine for risperidone restored the patient’s functionality within 24 hours, underscoring the importance of maintaining a high index of suspicion for drug-induced etiologies.

## Conclusions

This case highlights the diagnostic challenge of distinguishing medication-induced immobility from primary catatonia in psychiatric inpatients. In our patient, the combination of risperidone and supratherapeutic valproate produced profound sedation, tremors, and refusal to eat, closely mimicking catatonia. Rapid and complete clinical recovery occurred within 24 hours following discontinuation of risperidone and substitution with olanzapine, strongly supporting a drug-induced etiology and highlighting the reversibility of this presentation with appropriate medication adjustment.

Clinicians should maintain a high index of suspicion for medication-related adverse effects when new motor or behavioral changes emerge after initiation of antipsychotic therapy, particularly in patients receiving mood stabilizers such as valproate. Early recognition, careful monitoring of serum drug levels, and timely selection of alternative antipsychotics with fewer pharmacokinetic interactions may help prevent unnecessary diagnostic investigations and facilitate more rapid clinical recovery.
